# Cardiac left ventricular MRI texture analysis to derive texture characteristics of a healthy population: clinical implications

**DOI:** 10.1007/s00330-025-11848-y

**Published:** 2025-07-26

**Authors:** Ping Tie, Stephen J. Gandy, Jonathan Weir-McCall, John Graeme Houston

**Affiliations:** 1 Department of Imaging Science and Technology, School of Medicine, University of Dundee, Ninewells Hospital, Dundee, UK; 2 Department of Medical Imaging, School of Medicine, Shaoxing University, Shaoxing, China; 3 NHS Tayside Medical Physics, Ninewells Hospital, Dundee, UK; 4 Department of Cardiovascular Imaging, School of Biomedical Engineering and Imaging Sciences, King’s College London, London, UK; 5 NHS Tayside Department of Clinical Radiology, Ninewells Hospital, Dundee, UK

**Keywords:** Magnetic resonance imaging, cine, Image processing, computer-assisted, Myocardium, healthy volunteers

## Abstract

**Objectives:**

To examine the factors affecting radiomic analysis of the myocardium and to identify the optimal approach to image analysis.

**Materials and methods:**

Texture features were extracted from left ventricular (LV) non-contrast cine images. Texture variable reduction work was performed by studying the region of interest (ROI) size influence and intra- and inter-observer repeatability. The texture analysis (TA) process was applied to a healthy population (*n* = 600 individuals) for further analysis. ROI were defined at end diastole (ED) and end systole (ES), with separate ROI drawn encompassing the whole myocardial wall, septal wall, and the free wall. Differences between ED and ES; females and males; different ages; and myocardial regions were compared.

**Results:**

Fifteen TA variables were tested; the majority were affected by the phase of imaging. Texture varied significantly with age, with ED images and with a whole wall ROI best differentiating between age groups. In the detection of physiological differences between men and women, the combination of ED with whole myocardium quantification resulted in a greater number of features showing a significant difference between the sexes. Consistent TA results were obtained for ROIs placed on the whole myocardial wall and on the septal wall.

**Conclusions:**

In a cohort of healthy volunteers, we have identified a subset of features with high reproducibility that are invariant to the region of interest size. Use of the whole myocardium at ED results in the greatest discrimination of physiological changes associated with age and sex.

**Key Points:**

***Question*** *The characteristics of healthy population myocardium texture using cardiac MR texture analysis are unclear.*

***Findings*** *A reasonable number of robust texture variables can discriminate physiological changes associated with age and sex.*

***Clinical relevance*** *This work develops a deeper understanding of healthy population myocardium variability, thus helping to increase awareness and recognition of myocardial disease. It is also achievable without the need for contrast agent injections or additional scanning.*

**Graphical Abstract:**

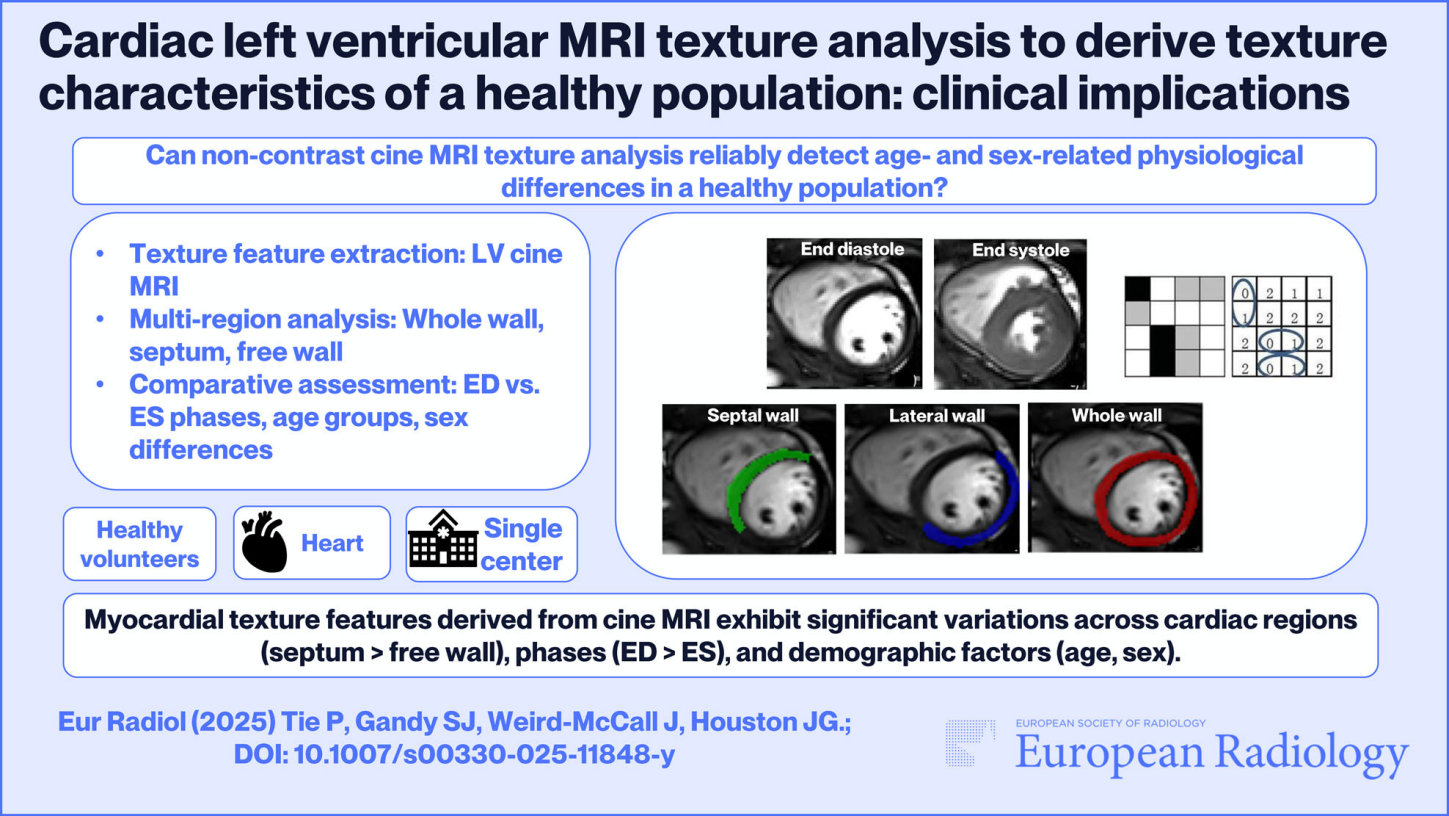

## Introduction

Cardiac magnetic resonance imaging is the reference standard for cardiac volumes and mass [1, 2]. It can accurately quantify the structure and function of the left ventricle, which is critical to clinical decision-making. Cine MRI is the most commonly used cardiac sequence, being ubiquitous within clinical protocols [3]. The traditional use of cine sequences is to assess the cardiac volumes, mass, and function, but there is potentially significant extra textural information in the images that may be closely related to tissue histology and pathology.

Texture analysis (TA) is a subgroup of radiomics that measures the non-uniformity of an image by the quantification of pixel greyscale variations. TA has seen widespread usage, especially in oncology, where greater tumor heterogeneity is associated with higher grade malignancy and poorer progression-free survival [4]. In cardiac MR, TA has shown feasibility in detecting viable/nonviable myocardium and differentiating between acute and chronic myocardial changes [5–8]. Several studies have demonstrated the ability of TA to distinguish cardiomyopathies [9–12] and myocarditis [13] from healthy controls. TA combined with T1 and T2 mapping has also been studied [11, 13–15]. In the UK Biobank, texture features were shown to be related to the presence of cardiovascular risk factors [16]. However, TA has been shown to be highly influenced by multiple factors [4, 17], and suffers from high dimensionality due to the hundreds of features it produces, with approaches to the understanding and mitigation of these issues in cardiac MRI poorly studied [18].

The objective of our research was therefore to develop the following strategies using routinely acquired non-contrast cine MR images: (1) to identify robust reproducible features that provide information on texture, and (2) to ascertain the optimal approach for radiomic analysis that best distinguishes between physiological changes associated with age and sex.

## Materials and methods

### Study population and process

The Tayside Screening for Cardiovascular Events (TASCFORCE) (REC Ref 07/S1402/42) study is a UK local population-based study involving healthy participants aged ≥ 40 years, with low-intermediate cardiovascular risk, and free of clinically apparent cardiovascular disease. All subjects included in this study had a cardiac MRI examination during the period 2008 to 2013 [19] and had demonstrated normal cardiac MR imaging findings. Each participant provided informed consent, and the project was approved by the ethical committee of the National Health Service (NHS) Tayside (REC Ref: 19/YH/0440).

### Image protocol

The cardiac MR protocol details have been published previously [19]. In brief, all images were obtained from the same 3-T scanner (Magnetom Trio, Siemens) using two RF coils (body array and spine array) for signal detection. A 2D-ECG-gated Cine TrueFISP sequence was used to acquire short-axis images of the heart (thickness = 6 mm, interslice gap = 4 mm, TR/TE = 3.4/1.5 ms, flip angle > 50°, pixel matrix = 173 × 256). Mid short-axis Cine slices of the left ventricle, at end diastole and end systole, were extracted from each subject for further image analysis.

### Region of interest definition

**Fig. 1:**
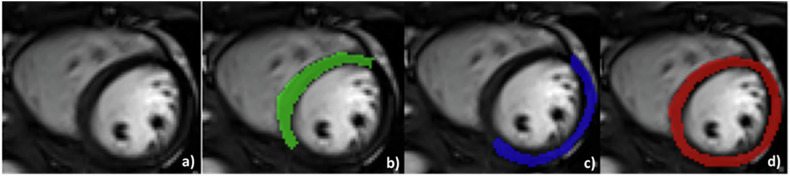
Different ROI placement, **a** original short-axis ED image; **b** ROI placed on the septal wall; **c** ROI placed on the lateral wall; **d** ROI including the whole short-axis myocardial wall. ROI, region of interest; ED, end-diastole

In order to explore possible myocardial regional differences, three different regions of interest (ROIs) were defined on the left ventricular myocardium for end-diastole (ED) and end-systole (ES) images: (1) an ROI placed on the septal wall; (2) an ROI on the free wall; and (3) an ROI including the whole mid short-axis section (see Fig. 1). This was conducted using MaZda v4.7. ROIs were all placed by observer 1 (P.T.), and for repeatability work, a second observer (S.G.) also completed the measurements. Both observers have > 10 years’ experience in the field of cardiac MR imaging.

### Statistical analysis

Statistical analysis was performed by SPSS software v 22.0. All data were tested for normality using the Shapiro–Wilk test. Data were presented as a mean ± standard deviation.

For comparisons, a paired *t*-test or Wilcoxon test was used for continuous variables, and independent samples *t*-test (otherwise Mann–Whitney test) or one-way ANOVA (otherwise Kruskal–Wallis test) for categorical variables as appropriate. A two-tailed *p*-value < 0.05 was considered statistically significant.

For correlations, a Spearman correlation was used for the strength of correlation assessment, and a *p*-value < 0.01 was considered statistically significant when using on texture features’ correlation results, where an absolute value of |r| < 0.5 indicated low correlation, 0.5 ≤ |r| < 0.8 intermediate correlation and 0.8 ≤ |r| strong correlation.

### Part I: Texture feature extraction and reduction

TA features for all images were derived and computed using MaZda. Gray-level normalization was executed with the TA software by adjusting the histogram data to fit within μ ± 3σ (μ being the gray-level mean and σ the standard deviation) to minimize the impact from image contrast alteration and brightness [20, 21].

By default, MaZda provides an extensive series of TA features—a total of 286 features can be generated. In order to reduce these numbers, the following two feature reduction strategies were implemented:

Step 1: Reduction of texture features from *n* = 286 to *n* = 144. The reduction process excluded many wavelet features, and also certain features associated with shape, remaining focused only on those related to 2D image texture-based features.

Step 2: Feature directionality reduction: In a large number of texture features, MaZda produces values at four 45-degree intervals (0/45/90/135). In any variables that had angulation and direction dependency, we averaged these, the rationale for which was that the degree of image rotation would be dependent on the precise angle of image acquisition rather than representing a true anatomically based axis. Furthermore, in a circular ROI—such as in the whole ventricle analysis—every segment would be balanced out by its opposite counterpart. This followed a similar approach used by Bettina [9, 22]. By implementing this step, we reduced the number of TA variables from *n* = 144 to *n* = 50 (including first-order, second order, and high-order, six subset texture features: Histogram (9), Absolute Gradient (5), Run-length matrix (5), Co-occurrence matrix (22), Auto-regressive model (5), Wavelet transform (4)) (Supplementary Table S-1).

Using the remaining variables, two studies were undertaken in order to establish which of the remaining 50 TA variables were the most stable and repeatable.

#### Study 1—ROI size test

The purpose of this test was to examine whether the size of the generated ROI resulted in alterations to the texture features. Subjects with the most extreme left ventricular mass (LVM) sizes were selected to reflect the whole cohort (*n* = 10 with the lowest LVM, *n* = 10 with median LVM, and *n* = 10 with the highest LVM)—details in Supplementary Table S-2. As both sex and age are known to affect texture features [23], we adjusted for this by using only female studies, and matched the group ages so that the mean age of each subject subset was selected to be consistent. ED phase images were used for this analysis, with all three regional ROIs drawn on each of these.

#### Study 2—Repeatability test

To evaluate repeatability of the remaining TA features, we conducted a small pilot study where a subset of *n* = 30 young (age 44 ± 3 years) healthy volunteers were selected, details as in Supplementary Table S-3. Cardiac phases at ED and ES were studied, using the ‘whole wall’ ROI placement method (Fig. 1d).

The ROI analysis was undertaken by two observers (observer 1—P.T. and observer 2—S.G.). Intra- and inter-observer testing was done by repeating all the measurements twice. The intra-observer test was completed by observer 1 (a month apart between the two measurements), and the inter-observer test was done by observer 1 and observer 2, respectively. The relative standard deviation (RSD) and root mean square coefficient of variation (RMS CoV) were implemented as the repeatability indexes, with the RMS CoV as driver factor and RSD as cofactor.

The TA variables were considered repeatable if the RMS CoV for both intra-observer and inter-observer variation was within 10%, and if the RSD was within 30% across the whole cohort. Additional validation details for these texture variables are provided in Supplementary Table S-4.

### Part II: Texture analysis in a large healthy population

Another *n* = 600 healthy volunteers were selected from the original TASCFORCE MRI cohort. Participants were assigned to four different age categories (≤ 45 years, 46–54 years, 55–63 years, or ≥ 64 years), and each of the age categories was gender-matched as far as possible. The participants were selected based on their LVM sizes, where only those within the range (mean ± 1 SD) for each gender were included. This was done to minimize any potential variation of TA variables with the ROI sizes placed. Texture differences between ED and ES, between females and males, among ages and different regions of the myocardial wall were assessed.

## Results

### Part I: Texture feature extraction and reduction

#### Study 1—ROI size test

**Fig. 2:**
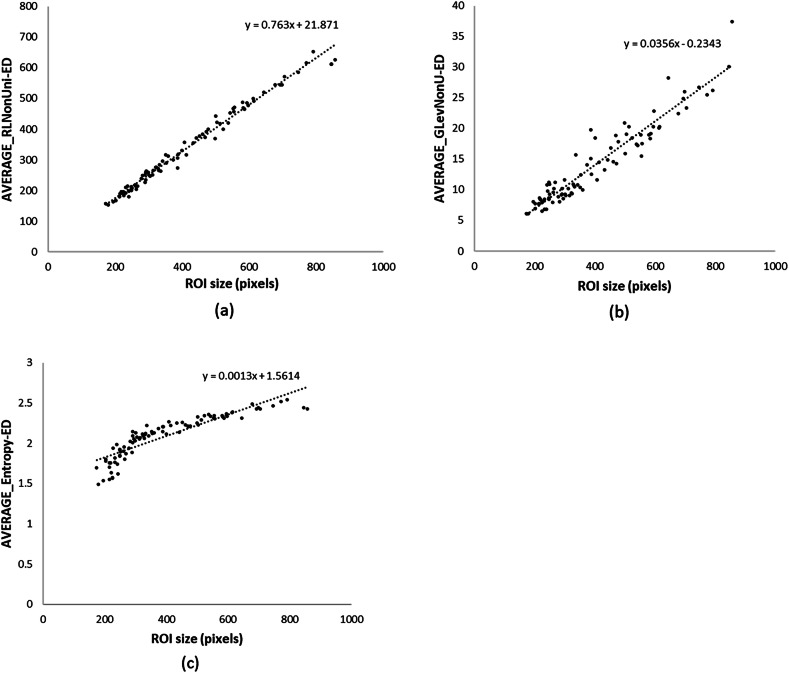
**a**–**c** Scatter plot showing three strongly correlated TA variables with ROI size variations. These TA variables were subsequently removed from further use in the main study

Although the three groups had significantly different ROI size ranges, most of the features were stable with ROI size variation. There were three exceptions which showed a clear correlation between ROI size and the TA value (|r| ≥ 0.80): *n* = 2 from the Run-length matrix family (Average_RLNonUni and Average_GLevNonU), and *n* = 1 from Co-ocurrence matrix family (S5-Average_Entropy) (see Fig. 2a–c). Because of their association with ROI size, these three TA features were excluded from the remainder of the study, resulting in *n* = 47 texture features to investigate further.

#### Study 2—Repeatability test

From the 47 TA variables tested, 15 (at ED) and 13 (at ES) were identified to be repeatable within and between observers, and also demonstrate an RSD within 20% across the whole cohort. These variables are highlighted in Table 1, representing those texture features to be used for the remainder of the study. Demographics of the test is in Supplementary Table S-3.

**Table 1:** Repeatability of texture variables (‘+’ means that the variable was able to fulfill the repeatability conditions)

Texture category	Texture feature	ED		ES	
		Intra-observer	Inter-observer	Intra-observer	Inter-observer
HISTOGRAM (0/9)		-	-	-	-
ABSOLUTE GRADIENT (2/5)	GrMean	+	+	+	+
	GrNonZeros	+	+	+	+
AUTOREGRESSIVE MODEL (0/5)		-	-	-	-
RUN LENGTH MATRIX (3/5)	AVERAGE_LngREmph	+	+	+	+
	AVERAGE_ShrtREmp	+	+	+	+
	AVERAGE_Fraction	+	+	+	+
COCURRENCE MATRIX—SHORT DISTANCES (S2) (5/11)	S2-AVERAGE_SumOfSqs	+	+	-	-
	S2-AVERAGE_SumAverg	+	+	+	+
	S2-AVERAGE_SumEntrp	**+**	+	**+**	**+**
	S2-AVERAGE_Entropy	**+**	+	**+**	**+**
	S2-AVERAGE_DifEntrp	**+**	+	**+**	**+**
COCURRENCE MATRIX—LONG DISTANCES (S5) (4/11)	S5-AVERAGE_InvDfMom	**+**	+	-	-
	S5-AVERAGE_SumAverg	**+**	+	**+**	**+**
	S5-AVERAGE_SumEntrp	**+**	+	**+**	**+**
	S5-AVERAGE_DifEntrp	**+**	+	**+**	**+**
WAVELET TRANSFORM (1/4)	AVERAGE_WavEnLL	**+**	+	**+**	**+**

*ED* end-diastole, *ES* end-systole

### Part II: Texture analysis in a large healthy population

#### Healthy population characteristics

The healthy volunteer cohort was comprised of 60% women and 40% men with a mean age of 55 ± 10 years. The four age groups were: group 1 ≤ 45 years (30% men), group 2 = 46–54 years (50% men), group 3 = 55–63 years (50% men), group 4 ≥ 64 years (32% men). The detailed demographic and cardiac MRI characteristics are provided in Table 2. As expected, female volunteers had lower body surface area, end-diastolic volume, end-systolic volume and left ventricular mass than male volunteers (*p* < 0.05).

**Table 2:** Healthy population characteristics

		Whole cohort	Age group 1 (≤ 45 years)	Age group 2 (46–54 years)	Age group 3 (55–63 years)	Age group 4 (≥ 64 years)	Age group differences
							(*p*-value)
Age range (years)	Whole (*n* = 600)	55.0 ± 9.9	43.2 ± 1.5	49.7 ± 2.5	58.7 ± 2.6	68.4 ± 4.3	
	Female (*n* = 357)	54.9 ± 10.5	43.1 ± 1.5	49.6 ± 2.6	58.6 ± 2.6	68.3 ± 4.1	
	Male (*n* = 243)	55.0 ± 9.2	43.3 ± 1.5	49.7 ± 2.5	58.8 ± 2.6	68.7 ± 4.5	
Body surface area (m²)	Whole (*n* = 600)	1.8 ± 0.2	1.8 ± 0.2	1.9 ± 0.2	1.9 ± 0.2	1.8 ± 0.2	0.01
	Female (*n* = 357)	1.7 ± 0.1	1.8 ± 0.2	1.7 ± 0.1	1.7 ± 0.1	1.7 ± 0.1	
	Male (*n* = 243)	2.0 ± 0.2	2.0 ± 0.2	2.0 ± 0.2	2.0 ± 0.1	2.0 ± 0.1	
Body mass index (kg/m²)	Whole (*n* = 600)	25.3 ± 3.6	25.7 ± 4.3	25.0 ± 3.8	25.4 ± 3.1	25.2 ± 3.3	0.41
	Female (*n* = 357)	25.5 ± 3.9	25.8 ± 4.5	25.2 ± 4.1	25.7 ± 3.5	25.4 ± 3.4	
	Male (*n* = 243)	25.0 ± 3.2	25.4 ± 3.8	24.8 ± 3.4	25.1 ± 2.6	24.8 ± 3.0	
Height (m)	Whole (*n* = 600)	1.69 ± 0.1	1.69 ± 0.1	1.71 ± 0.1	1.70 ± 0.1	1.67 ± 0.1	0.01
	Female (*n* = 357)	1.64 ± 0.1	1.65 ± 0.1	1.64 ± 0.1	1.63 ± 0.1	1.62 ± 0.1	
	Male (*n* = 243)	1.77 ± 0.1	1.78 ± 0.1	1.77 ± 0.1	1.76 ± 0.1	1.77 ± 0.1	
End diastolic volume (mL)	Whole (*n* = 600)	131.3 ± 28.8	135.8 ± 25.9	140.3 ± 29.6	129.5 ± 28.8	119.6 ± 26.9	0.00
	Female (*n* = 357)	116.8 ± 20.3	125.5 ± 19.6	121.6 ± 17.4	112.6 ± 20.1	107.5 ± 18.4	
	Male (*n* = 243)	152.5 ± 26.3	159.8 ± 23.0	159.0 ± 27.3	146.4 ± 26.3	145.2 ± 24.1	
End systolic volume (mL)	Whole (*n* = 600)	41.7 ± 14.3	44.3 ± 12.3	45.4 ± 13.8	40.2 ± 14.7	37.0 ± 14.6	0.00
	Female (*n* = 357)	36.4 ± 11.5	41.2 ± 11.1	37.6 ± 9.1	34.0 ± 11.7	32.2 ± 11.4	
	Male (*n* = 243)	49.6 ± 14.3	51.4 ± 12.1	53.1 ± 13.4	46.3 ± 15.0	47.4 ± 15.4	
Left ventricular mass (g)	Whole (*n* = 600)	101.0 ± 26.9	98.7 ± 27.1	107.3 ± 28.8	103.7 ± 27.4	94.4 ± 22.4	0.00
	Female (*n* = 357)	84.6 ± 15.3	85.4 ± 15.0	86.2 ± 15.5	83.4 ± 16.0	83.5 ± 14.9	
	Male (*n* = 243)	125.1 ± 21.6	129.7 ± 23.2	128.4 ± 23.1	123.9 ± 20.6	117.6 ± 17.3	
Ejection fraction (%)	Whole (*n* = 600)	68.6 ± 6.5	67.5 ± 5.9	67.8 ± 6.2	69.5 ± 6.7	69.7 ± 7.0	0.00
	Female (*n* = 357)	69.2 ± 6.7	67.3 ± 6.0	69.0 ± 6.3	70.2 ± 7.0	70.6 ± 7.0	
	Male (*n* = 243)	67.8 ± 6.2	67.9 ± 5.6	66.7 ± 5.9	68.7 ± 6.3	67.8 ± 6.8	

Values are presented as mean ± SD

#### Healthy population texture variables—differences between ED and ES

Table 3a, b illustrates variations of each of the *n* = 15 TA variables between ED and ES. There were *n* = 11 texture variables for ‘whole myocardial wall,’ *n* = 13 texture variables for ‘septal myocardial wall’ and *n* = 12 texture variables for ‘lateral myocardial wall,’ which changed between ED and ES (Table 3a). The ROI’s placed at the septal wall most consistently identified changes between systole and diastole compared with those placed elsewhere. This persisted when the analysis was stratified by gender and age category, with all the *n* = 13 texture variables showing significant differences between ED and ES in all groups (*p* < 0.05) (Table 3b).

**Table 3:** Variations of each of the *n* = 15 TA variables between ED and ES, where the data are presented for: **a** the full cohort, with different ROIs; **b** an ROI of the septal wall, with different gender and age groups

a									
	Whole wall ROI (full cohort)			Septal wall ROI (full cohort)			Lateral wall ROI (full cohort)		
	ED	ES	*p*-value	ED	ES	*p*-value	ED	ES	*p*-value
GrMean	2.79 ± 0.32	2.19 ± 0.31	0.00	2.97 ± 0.36	2.55 ± 0.41	0.00	3.14 ± 0.42	2.41 ± 0.41	0.00
GrNonZeros	0.92 ± 0.03	0.87 ± 0.05	0.00	0.93 ± 0.03	0.91 ± 0.04	0.00	0.94 ± 0.03	0.89 ± 0.05	0.00
AVERAGE_LngREmph	1.28 ± 0.08	1.42 ± 0.14	0.00	1.25 ± 0.08	1.31 ± 0.12	0.00	1.22 ± 0.08	1.34 ± 0.12	0.00
AVERAGE_ShrtREmp	0.95 ± 0.01	0.93 ± 0.01	0.00	0.95 ± 0.01	0.94 ± 0.02	0.00	0.96 ± 0.01	0.94 ± 0.02	0.00
AVERAGE_Fraction	0.93 ± 0.02	0.90 ± 0.02	0.00	0.93 ± 0.02	0.92 ± 0.02	0.00	0.94 ± 0.02	0.91 ± 0.02	0.00
S2-AVERAGE_SumOfSqs	85.08 ± 8.67	/	/	87.12 ± 8.86	/	/	92.39 ± 11.05	/	/
S2-AVERAGE_SumAverg	63.86 ± 0.63	64.57 ± 0.73	0.00	63.57 ± 0.75	65.03 ± 1.07	0.00	64.26 ± 0.75	64.99 ± 0.81	0.00
S2-AVERAGE_SumEntrp	1.69 ± 0.04	1.70 ± 0.06	0.01	1.62 ± 0.05	1.65 ± 0.06	0.00	1.67 ± 0.05	1.68 ± 0.06	0.00
S2-AVERAGE_Entropy	2.60 ± 0.07	2.60 ± 0.07	0.10	2.37 ± 0.08	2.44 ± 0.08	0.00	2.47 ± 0.09	2.49 ± 0.08	0.00
S2-AVERAGE_DifEntrp	1.32 ± 0.04	1.22 ± 0.06	0.00	1.31 ± 0.04	1.27 ± 0.06	0.00	1.34 ± 0.05	1.24 ± 0.07	0.00
S5-AVERAGE_InvDfMom	0.12 ± 0.02	/	/	0.09 ± 0.03	/	/	0.11 ± 0.02	/	/
S5-AVERAGE_SumAverg	65.03 ± 0.86	65.02 ± 0.82	0.69	63.94 ± 7.28	64.49 ± 1.47	0.00	64.71 ± 1.19	65.17 ± 1.16	0.00
S5-AVERAGE_SumEntrp	1.63 ± 0.04	1.67 ± 0.05	0.00	1.35 ± 0.16	1.57 ± 0.08	0.00	1.52 ± 0.07	1.6 ± 0.06	0.00
S5-AVERAGE_DifEntrp	1.36 ± 0.04	1.32 ± 0.05	0.00	1.19 ± 0.14	1.32 ± 0.06	0.00	1.31 ± 0.05	1.31 ± 0.06	0.22
AVERAGE_WavEnLL	27,111 ± 1778	22,518 ± 1680	0.00	32,139 ± 2105	26,744 ± 2298	0.00	24,928 ± 2506	19,825 ± 2145	0.00

b							
Septal wall ROI							
		Males (*n* = 243)	Females (*n* = 357)	Age group 1 (*n* = 150)	Age group 2 (*n* = 150)	Age group 3 (*n* = 150)	Age group 4 (*n* = 150)
			ED	ED	ED	ED	ED
GrMean	ED	2.82 ± 0.35	3.06 ± 0.33	3.08 ± 0.36	2.94 ± 0.34	2.90 ± 0.36	2.95 ± 0.34
	ES	2.41 ± 0.41	2.64 ± 0.37	2.65 ± 0.39	2.54 ± 0.38	2.45 ± 0.42	2.54 ± 0.41
GrNonZeros	ED	0.93 ± 0.03	0.94 ± 0.03	0.94 ± 0.03	0.94 ± 0.03	0.93 ± 0.03	0.93 ± 0.03
	ES	0.91 ± 0.04	0.92 ± 0.04	0.92 ± 0.04	0.91 ± 0.04	0.90 ± 0.04	0.91 ± 0.05
AVERAGE_LngREmph	ED	1.26 ± 0.09	1.23 ± 0.08	1.23 ± 0.09	1.25 ± 0.07	1.25 ± 0.08	1.25 ± 0.08
	ES	1.34 ± 0.13	1.29 ± 0.10	1.29 ± 0.09	1.31 ± 0.12	1.34 ± 0.12	1.31 ± 0.13
AVERAGE_ShrtREmp	ED	0.95 ± 0.01	0.95 ± 0.01	0.96 ± 0.01	0.95 ± 0.01	0.95 ± 0.01	0.95 ± 0.01
	ES	0.94 ± 0.02	0.95 ± 0.01	0.95 ± 0.01	0.94 ± 0.01	0.94 ± 0.02	0.94 ± 0.02
AVERAGE_Fraction	ED	0.93 ± 0.02	0.94 ± 0.02	0.94 ± 0.02	0.93 ± 0.02	0.93 ± 0.02	0.93 ± 0.02
	ES	0.91 ± 0.02	0.92 ± 0.02	0.92 ± 0.02	0.92 ± 0.02	0.91 ± 0.02	0.92 ± 0.02
S2-AVERAGE_SumOfSqs	ED	84.59 ± 8.87	88.84 ± 8.42	90.47 ± 8.32	86.80 ± 8.09	85.21 ± 8.75	85.98 ± 9.39
	ES	/	/	/	/	/	/
S2-AVERAGE_SumAverg	ED	63.62 ± 0.79	63.54 ± 0.73	63.80 ± 0.71	63.54 ± 0.74	63.47 ± 0.78	63.47 ± 0.74
	ES	64.90 ± 1.06	65.12 ± 1.08	65.06 ± 1.11	65.03 ± 1.01	64.93 ± 1.13	65.11 ± 1.04
S2-AVERAGE_SumEntrp	ED	1.64 ± 0.04	1.61 ± 0.04	1.62 ± 0.05	1.63 ± 0.05	1.63 ± 0.05	1.62 ± 0.05
	ES	1.67 ± 0.06	1.64 ± 0.06	1.66 ± 0.05	1.66 ± 0.06	1.65 ± 0.07	1.65 ± 0.06
S2-AVERAGE_Entropy	ED	2.42 ± 0.07	2.34 ± 0.07	2.35 ± 0.08	2.37 ± 0.08	2.39 ± 0.08	2.37 ± 0.08
	ES	2.47 ± 0.07	2.41 ± 0.07	2.43 ± 0.07	2.45 ± 0.07	2.44 ± 0.09	2.43 ± 0.08
S2-AVERAGE_DifEntrp	ED	1.30 ± 0.04	1.32 ± 0.04	1.32 ± 0.04	1.31 ± 0.04	1.31 ± 0.04	1.31 ± 0.04
	ES	1.25 ± 0.07	1.28 ± 0.06	1.28 ± 0.06	1.27 ± 0.06	1.25 ± 0.07	1.26 ± 0.06
S5-AVERAGE_InvDfMom	ED	0.10 ± 0.03	0.09 ± 0.03	0.09 ± 0.03	0.09 ± 0.03	0.10 ± 0.03	0.10 ± 0.02
	ES	/	/	/	/	/	/
S5-AVERAGE_SumAverg	ED	65.16 ± 4.77	63.10 ± 8.48	63.6 ± 8.40	62.96 ± 7.62	64.80 ± 5.33	64.39 ± 7.37
	ES	64.46 ± 1.08	64.51 ± 1.67	64.59 ± 2.03	64.42 ± 1.35	64.46 ± 1.09	64.50 ± 1.22
S5-AVERAGE_SumEntrp	ED	1.44 ± 0.13	1.29 ± 0.15	1.29 ± 0.15	1.35 ± 0.16	1.40 ± 0.15	1.37 ± 0.15
	ES	1.61 ± 0.06	1.54 ± 0.08	1.55 ± 0.09	1.58 ± 0.08	1.58 ± 0.08	1.57 ± 0.08
S5-AVERAGE_DifEntrp	ED	1.26 ± 0.11	1.15 ± 0.13	1.14 ± 0.13	1.19 ± 0.14	1.23 ± 0.13	1.21 ± 0.13
	ES	1.33 ± 0.06	1.32 ± 0.07	1.32 ± 0.07	1.33 ± 0.06	1.32 ± 0.06	1.32 ± 0.06
AVERAGE_WavEnLL	ED	31,485 ± 1947	32,584 ± 2092	32,405 ± 2230	32,373 ± 1854	31,921 ± 1987	31,857 ± 2287
	ES	26,328 ± 2191	27,027 ± 2326	27,310 ± 2599	27,091 ± 2105	26,286 ± 2171	26,288 ± 2121

The data are presented as mean + SD, with colored gray cells indicating *p* > 0.05; *p*-values for **b** are all < 0.05. The symbol ‘/’ indicates that the texture parameter is not consistently repeatable at ES, and therefore not calculated at ES. Full cohort *n* = 600 healthy volunteers

#### Healthy population texture variables—differences between females and males

From the selected 15 TA variables used in the study, there were *n* = 14 ‘whole wall,’ *n* = 13 ‘septal wall’ and *n* = 12 ‘lateral wall’ variables that could identify gender differences on ED images (Table 4a).

**Table 4:** Variations of each of the *n* = 15 TA variables between males and females, where the data are presented for the whole wall, the septal wall, and the lateral wall, at the different cardiac phases ED and ES: **a** at end-diastole, **b** at end-systole

a									
	ED								
	Whole wall			Septal wall			Lateral wall		
	Females (*n* = 357)	Males (*n* = 243)	*p*-value	Females (*n* = 357)	Males (*n* = 243)	*p*-value	Females (*n* = 357)	Males (*n* = 243)	*p*-value
GrMean	2.88 ± 0.31	2.65 ± 0.30	0.00	3.06 ± 0.33	2.82 ± 0.35	0.00	3.22 ± 0.42	3.03 ± 0.38	0.00
GrNonZeros	0.93 ± 0.02	0.91 ± 0.03	0.00	0.94 ± 0.03	0.93 ± 0.03	0.01	0.95 ± 0.03	0.94 ± 0.03	0.00
AVERAGE_LngREmph	1.27 ± 0.07	1.30 ± 0.08	0.00	1.23 ± 0.08	1.26 ± 0.09	0.00	1.22 ± 0.08	1.22 ± 0.07	0.02
AVERAGE_ShrtREmp	0.95 ± 0.01	0.94 ± 0.01	0.00	0.95 ± 0.01	0.95 ± 0.01	0.00	0.96 ± 0.01	0.96 ± 0.01	0.00
AVERAGE_Fraction	0.93 ± 0.01	0.92 ± 0.02	0.00	0.94 ± 0.02	0.93 ± 0.02	0.00	0.94 ± 0.02	0.94 ± 0.02	0.00
S2-AVERAGE_SumOfSqs	86.71 ± 8.35	82.68 ± 8.60	0.00	88.84 ± 8.42	84.59 ± 8.87	0.00	93.42 ± 10.32	90.88 ± 11.91	0.03
S2-AVERAGE_SumAverg	63.88 ± 0.62	63.83 ± 0.64	0.35	63.54 ± 0.73	63.62 ± 0.79	0.29	64.22 ± 0.74	64.33 ± 0.78	0.10
S2-AVERAGE_SumEntrp	1.69 ± 0.04	1.70 ± 0.04	0.00	1.61 ± 0.04	1.64 ± 0.04	0.00	1.66 ± 0.05	1.68 ± 0.05	0.00
S2-AVERAGE_Entropy	2.58 ± 0.06	2.63 ± 0.06	0.00	2.34 ± 0.07	2.42 ± 0.07	0.00	2.43 ± 0.08	2.53 ± 0.07	0.00
S2-AVERAGE_DifEntrp	1.33 ± 0.04	1.30 ± 0.05	0.00	1.32 ± 0.04	1.30 ± 0.04	0.00	1.34 ± 0.05	1.33 ± 0.05	0.01
S5-AVERAGE_InvDfMom	0.12 ± 0.02	0.13 ± 0.02	0.00	0.09 ± 0.03	0.10 ± 0.03	0.00	0.11 ± 0.02	0.11 ± 0.02	0.11
S5-AVERAGE_SumAverg	65.19 ± 0.85	64.80 ± 0.81	0.00	63.10 ± 8.48	65.16 ± 4.77	0.33	64.77 ± 1.18	64.61 ± 1.20	0.05
S5-AVERAGE_SumEntrp	1.62 ± 0.04	1.65 ± 0.04	0.00	1.29 ± 0.15	1.44 ± 0.13	0.00	1.50 ± 0.07	1.57 ± 0.06	0.00
S5-AVERAGE_DifEntrp	1.35 ± 0.04	1.36 ± 0.04	0.04	1.15 ± 0.13	1.26 ± 0.11	0.00	1.30 ± 0.05	1.33 ± 0.05	0.00
AVERAGE_WavEnLL	27,628 ± 1633	26,354 ± 1716	0.00	32,584 ± 2092	31,485 ± 1947	0.00	25,456 ± 2401	24,153 ± 2460	0.00

b									
	ES								
	Whole wall			Septal wall			Lateral wall		
	Females (*n* = 357)	Males (*n* = 243)	*p*-value	Females (*n* = 357)	Males (*n* = 243)	*p*-value	Females (*n* = 357)	Males (*n* = 243)	*p*-value
GrMean	2.28 ± 0.29	2.05 ± 0.29	0.00	2.64 ± 0.37	2.41 ± 0.41	0.00	2.51 ± 0.39	2.26 ± 0.39	0.00
GrNonZeros	0.88 ± 0.04	0.86 ± 0.05	0.00	0.92 ± 0.04	0.91 ± 0.04	0.00	0.90 ± 0.04	0.88 ± 0.05	0.00
AVERAGE_LngREmph	1.39 ± 0.11	1.47 ± 0.15	0.00	1.29 ± 0.10	1.34 ± 0.13	0.00	1.32 ± 0.10	1.39 ± 0.14	0.00
AVERAGE_ShrtREmp	0.93 ± 0.01	0.92 ± 0.02	0.00	0.95 ± 0.01	0.94 ± 0.02	0.00	0.94 ± 0.01	0.93 ± 0.02	0.00
AVERAGE_Fraction	0.90 ± 0.02	0.89 ± 0.02	0.00	0.92 ± 0.02	0.91 ± 0.02	0.00	0.92 ± 0.02	0.90 ± 0.03	0.00
S2-AVERAGE_SumOfSqs	/	/	/	/	/	/	/	/	/
S2-AVERAGE_SumAverg	64.56 ± 0.76	64.60 ± 0.69	0.42	65.12 ± 1.08	64.90 ± 1.06	0.02	64.96 ± 0.83	65.02 ± 0.79	0.42
S2-AVERAGE_SumEntrp	1.69 ± 0.06	1.71 ± 0.06	0.00	1.64 ± 0.06	1.67 ± 0.06	0.00	1.67 ± 0.05	1.69 ± 0.06	0.00
S2-AVERAGE_Entropy	2.59 ± 0.08	2.61 ± 0.07	0.00	2.41 ± 0.07	2.47 ± 0.07	0.00	2.47 ± 0.07	2.52 ± 0.08	0.00
S2-AVERAGE_DifEntrp	1.24 ± 0.05	1.20 ± 0.06	0.00	1.28 ± 0.06	1.25 ± 0.07	0.00	1.26 ± 0.06	1.22 ± 0.07	0.00
S5-AVERAGE_InvDfMom	/	/	/	/	/	/	/	/	/
S5-AVERAGE_SumAverg	65.07 ± 0.82	64.95 ± 0.81	0.08	64.51 ± 1.67	64.46 ± 1.08	0.74	65.21 ± 1.15	65.11 ± 1.19	0.22
S5-AVERAGE_SumEntrp	1.66 ± 0.05	1.69 ± 0.05	0.00	1.54 ± 0.08	1.61 ± 0.06	0.00	1.58 ± 0.06	1.63 ± 0.06	0.00
S5-AVERAGE_DifEntrp	1.33 ± 0.05	1.30 ± 0.05	0.00	1.32 ± 0.07	1.33 ± 0.06	0.00	1.31 ± 0.05	1.31 ± 0.06	0.38
AVERAGE_WavEnLL	22,714 ± 1718	22,229 ± 1587	0.00	27,027 ± 2326	26,328 ± 2191	0.00	19,980 ± 2217	19,598 ± 2017	0.02

Results presented as mean + SD, with colored gray cells indicating *p* > 0.05. The symbol ‘/’ indicates that the texture parameter is not consistently repeatable at ES, and therefore not calculated at ES

Similarly, there were *n* = 11 ‘whole wall,’ *n* = 12 ‘septal wall,’ and *n* = 10 ‘lateral wall’ variables that could identify gender differences on ES images (Table 4b).

#### Healthy population texture variables—differences between age groups

Age group TA differences were not as apparent as those between ED vs ES and Females vs Males. The key finding from this sub-study (Table 5a, b) was that TA could discriminate between age marginally better at end-diastole, and when a whole wall ROI was used (as opposed to medial or lateral wall ROIs). It was particularly difficult to identify any significant TA differences with age at the ‘lateral wall.’

**Table 5:** Variations of each of the *n* = 15 TA variables between age groups, where the data are presented for the whole wall, at the different cardiac phases ED and ES: **a** at ED, **b** at ES

a										
ED-whole wall ROI (full cohort)										
	Group 1 ( ≤ 45 years)	Group 2 (46–54 years)	Group 3 (55–63 years)	Group 4 ( ≥ 64 years)	*p*-value					
					Group 1 vs Group 2	Group 1 vs Group 3	Group 1 vs Group 4	Group 2 vs Group 3	Group 2 vs Group 4	Group 3 vs Group 4
GrMean	2.84 ± 0.34	2.74 ± 0.31	2.73 ± 0.33	2.85 ± 0.29	0.00	0.00	0.93	0.78	0.00	0.00
GrNonZeros	0.93 ± 0.03	0.92 ± 0.03	0.91 ± 0.03	0.93 ± 0.03	0.00	0.00	1.00	1.00	0.02	0.00
AVERAGE_LngREmph	1.27 ± 0.08	1.29 ± 0.07	1.30 ± 0.08	1.27 ± 0.07	0.00	0.00	1.00	1.00	0.02	0.00
AVERAGE_ShrtREmp	0.95 ± 0.01	0.95 ± 0.01	0.95 ± 0.01	0.95 ± 0.01	0.00	0.00	1.00	1.00	0.02	0.01
AVERAGE_Fraction	0.93 ± 0.02	0.92 ± 0.01	0.92 ± 0.02	0.93 ± 0.01	0.00	0.00	1.00	1.00	0.02	0.00
S2-AVERAGE_SumOfSqs	88.97 ± 8.18	83.77 ± 8.19	81.97 ± 8.29	85.60 ± 8.52	0.00	0.00	0.01	0.20	0.26	0.00
S2-AVERAGE_SumAverg	64.18 ± 0.63	63.75 ± 0.55	63.54 ± 0.52	63.97 ± 0.62	0.00	0.00	0.00	0.00	0.00	0.00
S2-AVERAGE_SumEntrp	1.70 ± 0.04	1.69 ± 0.04	1.69 ± 0.04	1.69 ± 0.04	1.00	0.01	0.58	0.20	1.00	0.71
S2-AVERAGE_Entropy	2.58 ± 0.07	2.62 ± 0.06	2.62 ± 0.06	2.60 ± 0.06	0.00	0.00	0.12	1.00	0.04	0.02
S2-AVERAGE_DifEntrp	1.32 ± 0.05	1.32 ± 0.04	1.31 ± 0.05	1.33 ± 0.04	/	/	/	/	/	/
S5-AVERAGE_InvDfMom	0.12 ± 0.02	0.12 ± 0.02	0.13 ± 0.02	0.12 ± 0.02	/	/	/	/	/	/
S5-AVERAGE_SumAverg	64.96 ± 0.87	65.11 ± 0.87	64.99 ± 0.83	65.06 ± 0.86	/	/	/	/	/	/
S5-AVERAGE_SumEntrp	1.61 ± 0.05	1.65 ± 0.04	1.65 ± 0.04	1.63 ± 0.04	0.00	0.00	0.26	1.00	0.00	0.00
S5-AVERAGE_DifEntrp	1.34 ± 0.04	1.36 ± 0.04	1.37 ± 0.04	1.36 ± 0.04	0.00	0.00	0.00	1.00	0.30	0.02
AVERAGE_WavEnLL	26,954 ± 1701	27,086 ± 1669	26,963 ± 1799	27,443 ± 1911	0.52	0.97	0.02	0.55	0.08	0.02

b										
ES-whole wall ROI (full cohort)										
	Group 1 ( ≤ 45 years)	Group 2 (46–54 years)	Group 3 (55–63 years)	Group 4 ( ≥ 64 years)	*p*-value					
					Group 1 vs Group 2	Group 1 vs Group 3	Group 1 vs Group 4	Group 2 vs Group 3	Group 2 vs Group 4	Group 3 vs Group 4
GrMean	2.22 ± 0.32	2.15 ± 0.30	2.14 ± 0.30	2.25 ± 0.33	0.06	0.03	0.35	0.73	0.00	0.00
GrNonZeros	0.88 ± 0.04	0.86 ± 0.04	0.86 ± 0.04	0.88 ± 0.05	0.01	0.00	1.00	1.00	0.07	0.00
AVERAGE_LngREmph	1.39 ± 0.13	1.44 ± 0.14	1.45 ± 0.13	1.41 ± 0.14	0.01	0.00	1.00	1.00	0.06	0.00
AVERAGE_ShrtREmp	0.93 ± 0.01	0.93 ± 0.02	0.92 ± 0.01	0.93 ± 0.01	0.01	0.00	1.00	1.00	0.20	0.00
AVERAGE_Fraction	0.90 ± 0.02	0.89 ± 0.02	0.89 ± 0.02	0.9 ± 0.02	0.00	0.00	1.00	1.00	0.10	0.00
S2-AVERAGE_SumOfSqs	/	/	/	/	/	/	/	/	/	/
S2-AVERAGE_SumAverg	64.66 ± 0.77	64.55 ± 0.70	64.45 ± 0.67	64.65 ± 0.77	0.20	0.01	0.94	0.23	0.23	0.02
S2-AVERAGE_SumEntrp	1.71 ± 0.06	1.70 ± 0.05	1.69 ± 0.06	1.70 ± 0.06	/	/	/	/	/	/
S2-AVERAGE_Entropy	2.59 ± 0.08	2.60 ± 0.07	2.60 ± 0.07	2.60 ± 0.08	/	/	/	/	/	/
S2-AVERAGE_DifEntrp	1.23 ± 0.06	1.22 ± 0.06	1.22 ± 0.06	1.24 ± 0.06	0.65	0.54	1.00	1.00	0.03	0.02
S5-AVERAGE_InvDfMom	/	/	/	/	/	/	/	/	/	/
S5-AVERAGE_SumAverg	65.34 ± 0.91	65.02 ± 0.83	64.81 ± 0.69	64.90 ± 0.71	0.00	0.00	0.00	0.02	0.20	0.31
S5-AVERAGE_SumEntrp	1.67 ± 0.05	1.68 ± 0.05	1.68 ± 0.05	1.67 ± 0.06	/	/	/	/	/	/
S5-AVERAGE_DifEntrp	1.32 ± 0.05	1.32 ± 0.04	1.32 ± 0.05	1.33 ± 0.05	/	/	/	/	/	/
AVERAGE_WavEnLL	22,342 ± 1700	22,558 ± 1658	22,551 ± 1690	22,620 ± 1682	/	/	/	/	/	/

Results presented as mean + SD, with colored gray cells indicating *p* > 0.05. The symbol ‘/’ indicates that the texture parameter is not consistently repeatable at ES, and therefore not calculated at ES. *N* = 150 subjects for each age group

## Discussion

This study was primarily designed to examine some of the fundamental aspects of 2D TA applied to non-contrast cine MR images of the LV myocardium. The work was performed at a single center and was standardized as much as possible by minimizing variations associated with the imaging factors, such as the same scanner, consistent MR protocol, image resolution, etc. By using this approach, our aim was to focus on searching for the highest power of each texture parameter to highlight differences associated with the myocardium, as opposed to being influenced by differences associated with the experimental conditions.

The original hypothesis of the work was to examine three different conditions, which we thought would influence the LV texture parameters by different amounts. Our expectation was that the LV TA variables would differ considerably with cardiac phase but be more stable with gender and age. This was largely borne out by our results (Tables 3–5), where mainly significant differences between the TA parameters were identified between those measured at ED versus ES, and those derived from males versus females. However, the TA parameters were more stable when it came to identifying differences between age groups, where the TA changes were considerably more subtle.

### Cine series

While TA has been shown in prior studies to differentiate cardiomyopathies and myocarditis from healthy controls—particularly when combined with mapping/late gadolinium enhancement (LGE)—we focused on cine images in this study for several reasons. First, this work was part of a larger population-based protocol assessing routine cardiac MR (CMR) variables alongside whole-body MR angiography (assessment of atherosclerotic plaque sites). Given that cine imaging is universally acquired in CMR, we aimed to rigorously evaluate TA on this widely available sequence, ensuring broader applicability compared to more specialized techniques (e.g., mapping or LGE). Our goal was to establish a foundational understanding of TA performance on standard cine data before progressing to advanced sequences. The study design (e.g., ED vs ES, male vs female, young vs old) remains equally valid for future TA applications in T1/T2 mapping or LGE—a logical next step.

### ROI size influence

It is known from previous studies that the ROI size can influence texture outcomes to some extent [4, 17, 24]. To investigate this further, we performed a small sub-study. By keeping the imaging parameters as consistent as possible, we were able to identify that most of the TA features tested were consistent with our ROI sizes, and those that were inconsistent were discarded from the rest of our work.

Of the *n* = 3 texture variables that were strongly correlated with ROI size, two of them were from the run-length matrix family (‘Average_RLNonUni’ and ‘Average_GLevNonU’), which calculates runs of pixels at a specific gray-level value in a definite orientation. The other variable was from the co-occurrence matrix-long distance family (‘S5-Average_Entropy’), which indicates the degree of disarray of the gray-level signal in the image. Similar results were also presented in a brain MRI TA study [25], where the authors hypothesized that the reason for ‘run-length matrix family’ variations is simply due to the fact that a larger ROI will result in more runs, or in the case of co-occurrence, more degrees of disarray. Of some concern, in many clinical studies of the utility of radiomics, gray-level non-uniformity has been one of the strongest texture features in assisting the separation of different myocardial disease [9, 26, 27]. Our work suggests these prior works should be interpreted with caution, as the difference between the groups may simply be picking up the difference in the degree of hypertrophy between the cohorts, rather than a unique texture fingerprint.

### ED versus ES

Texture variations between ED and ES images were hypothesized to be the largest; myocardial tissue pattern was quite different from each other between these two states [28]. The significant differences in myocardial texture between ED and ES observed during texture analysis may be attributed to several factors: (1) Myocardial thickening, strain, wall motion, and shear forces during the cardiac cycle alter the orientation and arrangement of myocardial fibers. These changes cause directional variations in tissue movement, leading to detectable texture differences [29]. (2) Intramyocardial blood flow: Perfusion differences between ED and ES affect local tissue contrast and texture patterns. (3) Stiffness and elasticity: The myocardium becomes stiffer during systole due to active contraction, whereas diastolic relaxation increases elasticity. These mechanical property changes influence texture characteristics [30]. (4) Imaging artifacts: Technique-dependent artifacts (e.g., in echocardiography) may also contribute to observed variations between ED and ES phases [31].

Previous studies that have reported cardiac MR texture analysis have preferred to focus on just one cardiac phase [6–8, 26, 32], while others have not mentioned the phase used, or have used the whole cardiac cycle of images [33]. Our findings confirm those of Alis et al [34], who examined radiomics in 59 healthy adults, and found that only 22 out of 352 showed strong stability over the cardiac cycle. Our study confirms these prior results, extending these by also showing that these differences hold true even after removing features associated with ROI size (which would be larger in systole). We also showed them to be present in a sex and age stratified analysis, as well as in considering the whole myocardium, or just the septal or free wall myocardium. The study highlights the importance of ensuring a consistent phase is used for radiomic analysis and warns against extrapolating findings from studies using systole to those using diastole.

### Female versus male

Gender differences are reported in studies that males may have a greater ‘physiological’ hypertrophy or bigger myocytes, along with greater myocyte cell loss with ageing [35–41]. In our study, we found significant differences in myocardial texture between men and women. Interestingly, we found that sex differences were present in the younger groups, but disappeared in the oldest group (≥ 64 years). A similar pattern has been observed in T1 mapping, where gender differences in native T1 are well described [40, 42]. In one study, while there is a significant gender difference in younger people (< 45 years, where T1 values in female myocardium are higher than those of males), in older age groups (≥ 45 years), this difference disappears [40]. These effects may reflect differences in the myocardium brought about by sex hormones, with these differences then becoming less pronounced in the post-menopausal period. Unfortunately, we do not have the menopausal status of the women in this study, which will require further study in future studies.

### Age group differences

Aging causes structural and functional changes of the heart, with measures such as myocardial triglyceride [43], cardiac fibrosis and cardiomyocyte hypertrophy are known to increase with age [41, 44–48]. In line with this, we have observed the texture features to change with age, which likely reflect several underlying physiological and microstructural changes. These features exhibit phase-dependent variability, with end-diastole providing the most reliable measurements. This temporal pattern primarily likely results from superior diastolic image quality, as the left ventricle achieves maximal volume and minimal motion during this quiescent phase of the cardiac cycle.

### Regional differences

From the ‘helical ventricular myocardial band of Torrent-Guasp’ [49], we know that the LV three-dimensional structure is different between the septal and lateral walls, where two layers of the fibers radiate in different directions [50]. Also, in hypertrophic cardiomyopathy (HCM) patients, cardiac pathological abnormalities such as fibrosis are more commonly found at the septum [51]. It is unknown whether the MR textural basis of the septum and the lateral wall is different in a healthy population, and there is therefore a need to understand this regionally specific texture further. In our study, we found that TA at the septal wall was more sensitive to variations between genders and age groups, while the lateral wall texture was more stable with less variation related to cardiac phase, gender, and age groups. This is likely a consequence of the lateral wall being most susceptible to artifacts, including susceptibility artifacts and chemical shift artifacts due to the close proximity of the epicardial fat and adjacent lung tissue. Similar findings have been shown in T1 mapping, where the septum produces the most reliable and reproducible region for tissue characterization. Consequently, we suggest a preferential use of the septal wall when appropriate for radiomic analysis. This approach may be particularly useful in the early stages of disease. However, the whole myocardium is also recommended as an ROI for analysis due to several advantages: (1) The septum was found to be superior in detecting change relative to the lateral wall, but this superiority was not as evident when compared to the whole myocardium, especially when examining subtle changes such as those related to age. (2) The whole myocardium ROI offers more reproducible segmentation (a capability available on most MR workstations), ensuring greater measurement robustness. This approach is also more clinically practical and provides comprehensive coverage of myocardial tissue—particularly valuable for diffuse cardiomyopathies where pathological changes may not be regionally confined.

### The recommended texture variables

There were *n* = 15 texture features that were identified to be repeatable and feasible for use in this study (Descriptions of all 15 texture features are available in Supplementary Material S-5). However, the most reliable parameters may not necessarily demonstrate effectiveness for all occasions (e.g., S2-AVERAGE_SumOfSqs was most useful for the identification of different genders and age groups but could not be used for cardiac phase differentiation). The use of these TA variables in clinical studies may be determined by the specific application and the cohort being studied.

In terms of measurement repeatability, the only previously published work is reported in [6]. The most important texture values they selected in their final study were Teta1 and Perc.01, which were excluded from our study. We found that these autoregressive model texture features (and also the histogram texture features) had very poor repeatability. These differences may be influenced by several aspects, including different field strength of magnet (1.5 T vs 3.0 T), MRI protocol, RF coils, etc.

Potential physiological mechanisms underlying these observed changes across different conditions primarily include: (1) Myocardial wall thickening alters partial volume effects, while perfusion changes modify signal intensity distribution, and myofibre structural reorganization impacts spatial tissue uniformity. (2) Increased microstructural complexity/disorganization, combined with mildly anisotropic fiber arrangement, influences both texture orientation patterns and signal characteristics. Illustrative Example: The parameter S2-AVERAGE_SumAverg demonstrates consistent statistical significance when comparing different age groups at ED. As this variable quantifies the sum of average gray-level intensities within local myocardial regions, its discriminative power likely captures: early extracellular matrix reorganization preceding clinically detectable fibrosis, subtle lipid deposition patterns that alter local signal averages, and microstructural changes associated with normal myocardial aging.

## Limitations

The current study was conducted on a single field strength on a single vendor. Replication of the findings in other settings is thus required. Second, we have taken a rigorous image feature-based approach to feature selection. Finally, while we proved texture variables could identify subtle myocardium differences in physiology and function, we do not have pre- and post-contrast T1 mapping available, which would allow for a deeper understanding of what changes in the myocardium are driving these shifts in the myocardial texture, with further work now required in this regard. Additionally, the lack of comprehensive databases for younger populations restricts the generalizability of our findings, highlighting the need for future studies to address this demographic specifically.

## Conclusion

In this cohort of healthy volunteers, we have identified a subset of texture features with high reproducibility that are invariant to ROI size. We also showed that the use of the whole myocardium at ED resulted in the greatest discrimination of physiological changes associated with age and sex. The combination of these outputs will aid in dimensionality reduction in radiomic analysis while ensuring the most sensitive approach to quantification is used.

## Supplementary information

Supplementary information

## Abbreviations

2D: 2-Dimentional
CMR: Cardiac Magnetic Resonance
ED: End-diastole
ES: End-systole
HCM: Hypertrophic Cardiomyopathy
LGE: Late gadolinium enhancement
LV: Left ventricular
LVM: Left ventricular mass
NHS: National Health Service
RMS CoV: Root mean square coefficient of variation
ROI: Region of interest
RSD: Relative standard deviation
TA: Texture analysis
TASCFORCE: Tayside Screening for Cardiovascular Events
