# Supplementary material for: Cardiac left ventricular MRI texture analysis to derive texture characteristics of a healthy population: clinical implications
Source: Eur Radiol. 2025 Jul 26;36(1):203–18. doi: 10.1007/s00330-025-11848-y (PMC12712044; doi:10.1007/s00330-025-11848-y)
Supplement: Supplementary file 1 — Supplementary information [file 330_2025_11848_MOESM1_ESM.pdf]

Cardiac left ventricular MRI texture analysis to derive texture characteristics of a healthy population: clinical implications

ELECTRONIC SUPPLEMENTARY MATERIAL

Supplementary Table S-1:

Table S- 1: N=50 'shortlisted' texture variables and the corresponding texture categories

Texture Type	Texture feature
HISTOGRAM（9）	Mean
	Variance
	Skewness
	Kurtosis
	Perc01
	Perc10
	Perc50
	Perc90
	Perc99
ABSOLUTE GRADIENT（5）	GrMean
	GrVariance
	GrSkewness
	GrKurtosis
	GrNonZeros
AUTOREGRESSIVE MODEL（5）	Teta1
	Teta2
	Teta3
	Teta4
	Sigma
RUN LENGTH MATRIX（5）	AVERAGE_RLNonUni
	AVERAGE_GLevNonU
	AVERAGE_LngREmph
	AVERAGE_ShrtREmp
	AVERAGE_Fraction
COCURRENCE MATRIX - SHORT DISTANCES (S2)（11）	AVERAGE_AngScMom
	AVERAGE_Contrast
	AVERAGE_Correlat

	AVERAGE_SumOfSqs
	AVERAGE_InvDfMom
	AVERAGE_SumAverg
	AVERAGE_SumVarnc
	AVERAGE_SumEntrp
	AVERAGE_Entropy
	AVERAGE_DifVarnc
	AVERAGE_DifEntrp
COCURRENCE MATRIX - LONG DISTANCES (S5)（11）	
	AVERAGE_AngScMom
	AVERAGE_Contrast
	AVERAGE_Correlat
	AVERAGE_SumOfSqs
	AVERAGE_InvDfMom
	AVERAGE_SumAverg
	AVERAGE_SumVarnc
	AVERAGE_SumEntrp
	AVERAGE_Entropy
	AVERAGE_DifVarnc
	AVERAGE_DifEntrp
WAVELET TRANSFORM（4）	AVERAGE_WavEnLL
	AVERAGE_WavEnLH
	AVERAGE_WavEnHL
	AVERAGE_WavEnHH

Supplementary Table S-2:

Table S-2 : Demographics and CMR left ventricular measurements of three different LVM groups (lower, medial, & higher). Data are presented by Mean ± SD. P-values for comparison among three different LVM groups, $p<0.05$ indicate statistical significance between the groups. ROI region of interest, SD standard deviation.

**Parameter**		**Lower group (n=10)**	**Median group (n=10)**	**Higher group (n=10)**	***P* value**
Gender		Female	Female	Female	
Age (years)		63.1±7.6	59.6±11.2	56.9±8.2	0.35
Weight (kg)		57.6±5.8	64.7±7.7	77.5±13.1	0
Height (cm)		157.3±5.5	163.2±4.7	168.5±8.0	0
Body Surface Area (m²)		1.6±0.1	1.7±0.1	1.9±0.2	0
Body Mass Index (kg/m²)		24.2±2.9	24.7±2.4	27.5±5.4	0.13
Heart rate (bpm)		67.2±9.8	71.3±5.2	68.6±9.3	0.54
Ejection Fraction (%)		70.1±9.5	64.8±7.1	68.5±12.2	0.47
End Diastolic Volume (ml)		90.7±18.3	116.5±14.4	135.8±11.8	0
End Systolic Volume (ml)		28.4±13.9	41.4±11.6	42.9±17.8	0.07
Left Ventricular Mass (g)		56.4±5.5	83.1±0.4	121.1±7.1	0
ROI (pixels)	whole wall	491±34	583±23	743±72	0
	septal wall	206±22	257±30	294±49	0
	lateral wall	269±37	327±31	427±54	0

Supplementary Table S-3:

Table S-3: Demographics of the repeatability test group. Results are presented by Mean ± SD.

**Parameter**	**Young group (n=30)**
Gender	15F: 15M
Age (years)	44.2±2.6
Weight (kg)	76.7±11.8
Height (cm)	170.4±11.3
Body Surface Area (m^2)	1.9±0.2
Body Mass Index (kg/m^2)	26.5±4.8
Heart rate (bpm)	63.0±10.0
Ejection Fraction (%)	67.0±5.2
End Diastolic Volume (ml)	143.9±25.7
End Systolic Volume (ml)	47.3±10.6
Left Ventricular Mass (g)	111.4±33.4

Supplementary Table S-4:

Following the repeatability analysis, additional validation of the preferred texture variables was performed, including the following assessments:

1)Influence of parameters on each other was evaluated using Spearman's correlation analysis.

As shown in *Table S-4(a-b),* several TA variables demonstrated mutual correlations (particularly between variables within expected 'families'). However, no single variable showed significant correlation with all other TA variables.

Table S-4: Correlation matrix of the n=15 TA variables, where the data are presented for the whole wall- at two cardiac phases ED and ES. a) ED phase; b) ES phase. Only correlations with absolute coefficients (|r|) ≥ 0.70 are displayed. Numeric identifiers in parentheses correspond to texture variables (e.g., (1) represents GrMean).

Spearman correlation (r)		(1)	(2)	(3)	(4)	(5)	(6)	(7)	(8)	(9)	(10)	(11)	(12)	(13)	(14)	(15)
(1)	GrMean-ED		0.78	-0.81	0.85	0.84					0.98	-0.73			0.84	
(2)	GrNonZero-ED	0.78		-0.89	0.87	0.90					0.77	-0.75				
(3)	AVERAGE_LngREmph-ED	-0.81	-0.89		-0.93	-0.99					-0.79	0.70				
(4)	AVERAGE_ShrtREmp-ED	0.85	0.87	-0.93		0.98					0.85	-0.76			0.72	
(5)	AVERAGE_Fraction-ED	0.84	0.90	-0.99	0.98						0.83	-0.74				
(6)	S2-AVERAGE_SumOfSqs-ED								0.84					0.71		
(7)	S2-AVERAGE_SumAverg-ED															
(8)	S2-AVERAGE_SumEntrp-ED						0.84			0.72				0.91		

	(1)	(2)	(3)	(4)	(5)	(6)	(7)	(8)	(9)	(10)	(11)	(12)	(13)	(14)	(15)
(9) S2-AVERAGE_Entropy-ED								0.72					0.74		
(10) S2-AVERAGE_DifEntrp-ED	0.98	0.77	-0.79	0.85	0.83						-0.76			0.87	
(11) S5-AVERAGE_InvDfMom-ED	-0.73	-0.75	0.70	-0.76	-0.74					-0.76				-0.81	
(12) S5-AVERAGE_SumAverg-ED															
(13) S5-AVERAGE_SumEntrp-ED						0.71		0.91	0.74						
(14) S5-AVERAGE_DifEntrp-ED	0.84			0.72						0.87	-0.81				
(15) AVERAGE_WavEnLL-ED															

a)

Spearman correlation (r)	(1)	(2)	(3)	(4)	(5)	(6)	(7)	(8)	(9)	(10)	(11)	(12)	(13)	(14)	(15)
(1) GrMean-ES		0.78	-0.81	0.85	0.84					0.98	-0.73			0.84	
(2) GrNonZeros-ES	0.78		-0.89	0.87	0.90					0.77	-0.75				
(3) AVERAGE_LngREmph-ES	-0.81	-0.89		-0.93	-0.99					-0.79	0.70				
(4) AVERAGE_ShrtREmp-ES	0.85	0.87	-0.93		0.98					0.85	-0.76			0.72	
(5) AVERAGE_Fraction-ES	0.84	0.90	-0.99	0.98						0.83	-0.74				
(6) S2-AVERAGE_SumOfSqs-ES								0.84					0.71		
(7) S2-AVERAGE_SumAverg-ES															

**(8)**	**S2-AVERAGE_SumEntrp-ES**						0.84			0.72				0.91		
**(9)**	**S2-AVERAGE_Entropy-ES**								0.72					0.74		
**(10)**	**S2-AVERAGE_DifEntrp-ES**	0.98	0.77	-0.79	0.85	0.83						-0.76			0.87	
**(11)**	**S5-AVERAGE_InvDfMom-ES**	-0.73	-0.75	0.70	-0.76	-0.74					-0.76				-0.81	
**(12)**	**S5-AVERAGE_SumAverg-ES**															
**(13)**	**S5-AVERAGE_SumEntrp-ES**						0.71		0.91	0.74						
**(14)**	**S5-AVERAGE_DifEntrp-ES**	0.84			0.72						0.87	-0.81				
**(15)**	**AVERAGE_WavEnLL-ES**															

b)

2) Parameter accuracy validation: The discriminatory power of extracted features was evaluated by assessing their ability to consistently differentiate between cardiac phases (ED vs. ES) across distinct age cohorts. As demonstrated in *Table S-4 (c)*, nearly all texture parameters successfully distinguished ED from ES images, with consistent performance observed in both age groups.

Supplemental Table S-4 c): Phase-dependent texture parameter variations. Statistical comparison of n=15 texture features between ED and ES phases (n=30 young and n=30 elderly subjects; $p<0.05$ indicates statistical significance). Measurements were obtained from whole-wall ROI analyses. Data presented as mean ± standard deviation. Cohort characteristics: Young group (age 68±4 years, 1:1 female:male ratio); Elderly group (age 68±4 years, 1:1 female:male ratio).

**Texture feature**	**ED**		**ES**		**P-value**	
	**Young**	**Old**	**Young**	**Old**	**ED v ES (Young)**	**ED v ES (old)**
**GrMean**	2.72±0.41	2.69±0.29	2.01±0.30	2.18±0.27	0.00	0.00
**GrNonZeros**	0.90±0.04	0.91±0.03	0.84±0.06	0.86±0.04	0.00	0.00
**AVERAGE_LngREmph**	1.33±0.17	1.30±0.08	1.53±0.27	1.44±0.13	0.00	0.00
**AVERAGE_ShrtREmp**	0.94±0.02	0.95±0.01	0.92±0.02	0.93±0.01	0.00	0.00
**AVERAGE_Fraction**	0.92±0.03	0.92±0.01	0.88±0.04	0.89±0.02	0.00	0.00
**S2-AVERAGE_SumOfSqs**	86.09±6.59	83.43±7.58	73.14±13.12	76.69±10.19	0.00	0.01
**S2-AVERAGE_SumAverg**	63.89±0.57	63.95±0.57	64.11±0.63	64.70±0.67	0.15	0.00
**S2-AVERAGE_SumEntrp**	1.69±0.04	1.69±0.03	1.68±0.07	1.70±0.05	0.65	0.47
**S2-AVERAGE_Entropy**	2.58±0.07	2.60±0.05	2.57±0.08	2.61±0.07	0.38	0.65
**S2-AVERAGE_DifEntrp**	1.31±0.06	1.31±0.04	1.19±0.07	1.23±0.05	0.00	0.00
**S5-AVERAGE_InvDfMom**	0.13±0.02	0.13±0.02	0.16±0.02	0.15±0.02	0.00	0.00
**S5-AVERAGE_SumAverg**	65.35±0.83	65.20±0.84	65.01±0.76	64.87±0.52	0.10	0.07
**S5-AVERAGE_SumEntrp**	1.63±0.05	1.64±0.03	1.66±0.05	1.68±0.05	0.01	0.00
**S5-AVERAGE_DifEntrp**	1.35±0.04	1.36±0.03	1.30±0.05	1.33±0.04	0.00	0.00
**AVERAGE_WavEnLL**	27483.72±2121.86	26814.14±1808.93	22388.07±1629.31	22612.56±1251.28	0.00	0.00

c) Following initial analysis, the robustness of the extracted TA variables was further validated

Supplementary material S-5: Fifteen texture variable descriptions (N=15)

GrMean (Gray-Level Mean) -Represents the average intensity value of all pixels within a specified ROI. Variations occur throughout the cardiac cycle or across spatial regions due to dynamic changes in myocardial thickness and partial volume effects.

GrNonZeros (Gray-Level Non-Zero Count) -Measures the number of non-zero pixel intensity values within a defined ROI. In cine MR, it provides insights into tissue heterogeneity and signal distribution.

AVERAGE_LngREmph (Average Local normalized Gray-Level Run-Length Emphasis)- Statistically quantifies the distribution of longer pixel runs (consecutive pixels with the same gray level). Emphasizes homogeneous tissue regions (long runs), microstructural orientation, and local gray-level variation patterns.

AVERAGE_ShrtREmp (Average Short Run Emphasis)- Specifically quantifies the prominence of short runs (few consecutive pixels with the same gray value). Reflects microscopic tissue heterogeneity, localized intensity abruptions, and structural complexity.

AVERAGE_Fraction - Measures the proportion of short runs relative to the total number of runs. Highlights regions where small, abrupt intensity variations dominate the texture pattern. Healthy myocardium exhibits aligned fibers, leading to longer runs.

S2-AVERAGE_SumOfSqs ***(****Average Sum of Squares****)*** - Represents the average of squared gray-level intensity sums within local regions, reflecting the degree of pixel intensity dispersion and local variability. Associated with tissue uniformity and heterogeneity.

S2-AVERAGE_SumAverg - Quantifies the sum of average gray-level intensities within local regions, evaluating the central tendency of overall signal intensity. Directly reflects the linear mean intensity of pixels.

S2-AVERAGE_SumEntrp (Average Sum of Entropy) - Calculates total texture randomness across all analyzed regions by summing local entropy values. Higher numbers indicate widespread tissue disorganization, helping detect diffuse diseases like fibrosis or amyloidosis.

S2-AVERAGE_Entropy - Measures randomness in pixel intensity variations within small image areas. High values indicate chaotic patterns (diseased tissue), while low values suggest uniform structure (healthy muscle).

S2-AVERAGE_DifEntrp (Average_Difference Entropy) - Measures directional variations in tissue texture by comparing entropy across orientations. Higher values reveal abnormal structural asymmetry, indicating disrupted fiber alignment in conditions like infarction.

S5-AVERAGE_InvDfMom ***(***Average Inverse Difference Moment***)*** - Measures smoothness of pixel intensity transitions. Higher values indicate uniform tissue

(healthy muscle), while lower values reveal rough textures (scarred areas). Acts as a "smoothness detector."

S5-AVERAGE_SumAverg-Measures the total accumulated brightness of local areas by summing their average intensities - higher values indicate stronger overall signal (like edema) while lower values suggest signal loss (e.g., iron deposits).

S5-AVERAGE_SumEntrp - Sums local randomness values across the entire image. Elevated totals indicate globally disorganized tissue patterns (e.g., diffuse fibrosis). Functions as a "whole-image chaos meter."

S5-AVERAGE_DifEntrp - Quantifies directional differences in local randomness. Increased values reveal unevenly disordered tissue (characteristic of infarct border zones), helping identify transitional areas.

AVERAGE_WavEnLL ***(***Average Wavelet Energy – Low-Low Subband***)*** - Calculates energy in the smoothest wavelet subband. Reduced values indicate loss of organized structure (e.g., myofiber disruption), serving as a "macro-pattern detector."
